# Burnt Plastic (Pyroplastic) from the M/V *X-Press Pearl* Ship Fire and Plastic Spill Contain Compounds That Activate Endocrine and Metabolism-Related Human and Fish Transcription Factors

**DOI:** 10.1021/envhealth.4c00172

**Published:** 2024-10-30

**Authors:** Bryan D. James, Alexander V. Medvedev, Lyubov A. Medvedeva, Elena Martsen, Kristen L. Gorman, Benjamin Lin, Sergei S. Makarov, Lihini I. Aluwihare, Asha de Vos, Christopher M. Reddy, Mark E. Hahn

**Affiliations:** † Department of Marine Chemistry and Geochemistry, Woods Hole Oceanographic Institution, Woods Hole, Massachusetts 02543, United States; ‡ Department of Biology, Woods Hole Oceanographic Institution, Woods Hole, Massachusetts 02543, United States; § Department of Chemical Engineering, Northeastern University, Boston, Massachusetts 02115, United States; ∥ Attagene, Research Triangle Park, Morrisville, North Carolina 27709, United States; ⊥ Scripps Institution of Oceanography, University of California San Diego, La Jolla, California 92093, United States; # Oceanswell, Colombo 00500, Sri Lanka; ∇ The Oceans Institute, University of Western Australia, Perth 6009, Australia

**Keywords:** nurdle, pollution, microplastic, open burning, maritime accident, bioactivity

## Abstract

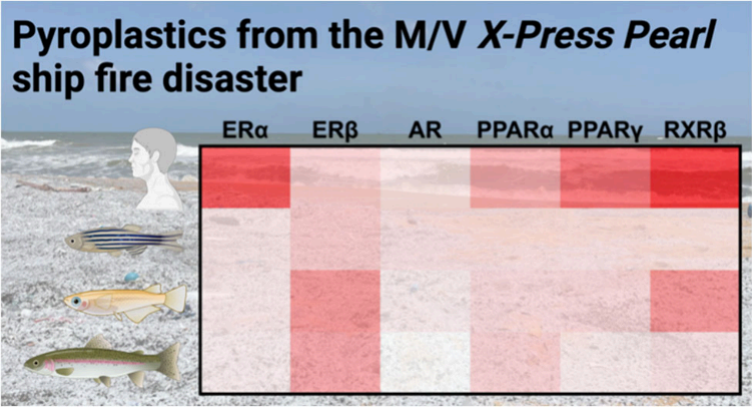

In May 2021, the M/V *X-Press Pearl* ship fire disaster led to the largest maritime spill of resin pellets (nurdles) and burnt plastic (pyroplastic). Field samples collected from beaches in Sri Lanka nearest to the ship comprised nurdles and pieces of pyroplastic. Three years later, the toxicity of the spilled material remains unresolved. To begin understanding its potential toxicity, solvent extracts of the nurdles and pyroplastic were screened for their bioactivity by several Attagene FACTORIAL bioassays (TF, NR, and AquaTox), which measured the activity of a combined 70 human transcription factor response elements and nuclear receptors and 6 to 7 nuclear receptors for each of three phylogenetically distinct fish species. Extracts of the pyroplastics robustly activated end points for the human aryl hydrocarbon receptor (AhR), estrogen receptor (ER), pregnane X receptor (PXR), peroxisome proliferator-activated receptor (PPAR), retinoid X receptor (RXR), and oxidative stress (NRF2) and had the potential for activation of several others. The bioactivity profile of the pyroplastics was most similar (similarity score = 0.96) to that of probable human carcinogens benzo[*b*]fluoranthene and benzo[*k*]fluoranthene despite the extracts being a complex mixture of thousands of compounds. The activity diminished only slightly for extracts of pyroplastic collected eight months after the spill. The AquaTox FACTORIAL bioassay measured the activation of ERα, ERβ, androgen receptor (AR), PPARα, PPARγ, and RXRβ for human, zebrafish (*Danio rerio*), Japanese medaka (*Oryzias latipes*), and rainbow trout (*Oncorhynchus mykiss*), revealing species-specific sensitivities to the chemicals associated with the pyroplastics. These findings provide needed information to guide long-term monitoring efforts, make hazard assessments of the spilled material, and direct further research on pyroplastic, an emerging global contaminant.

## Introduction

In late May 2021, off the coast of Colombo, Sri Lanka, the ship fire and subsequent plastic spill of the M/V *X-Press Pearl* released ∼1680 tons of plastic nurdles and other plastic debris, making it the largest maritime plastic spill in history.^1^ Along with polyethylene pellets, the cargo on the ship included an assortment of raw materials, hazardous chemicals, and finished products,^2^ capable of creating a complex mixture of uncertain toxicity. An observable fraction of the spilled material included burnt plastic (pyroplastic),^2,3^ formed during the events of the ship fire. The pyroplastic was heterogeneous in size and shape and somewhat friable, giving it a greater propensity to form secondary microplastics than the other spilled material.^2,4^ The attributes of the pyroplastic collectively challenged the response efforts and elevated the plastic’s potential for injury to a host of marine organisms.^1,2^

**Figure 1:**
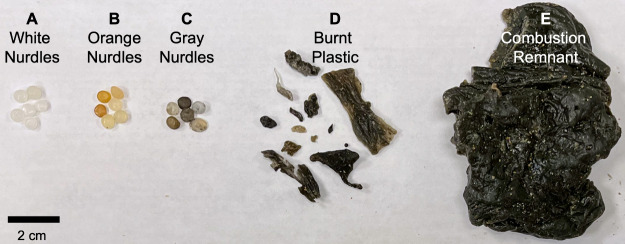
Spilled plastic included white nurdles (A), orange nurdles (B), gray nurdles (C), pieces of burnt plastic (D), and larger combustion remnant chunks (E). Reprinted from James et al.^5^ (CC BY-NC-ND 4.0).

At least five forms of plastic were released, including three types of nurdles distinguished by their color (white, orange, and gray) and two types of pyroplastic characterized by their shape and size (burnt plastic and combustion remnants) (Figure 1).^5,6^ Pieces of pyroplastic were not only at least 3-fold more chemically complex because of the fire,^2^ they were shown to have the greatest content of polycyclic aromatic hydrocarbons (PAHs) of any plastic marine debris recorded to date, 199,000 ng/g.^5^ Comparatively, the more abundant white nurdles had PAH contents less than ∼2500 ng/g, within the range of other marine debris.^5^ PAHs are chemical pollutants, many of which are carcinogenic, raising concern over the release of pyroplastic into the environment. While substantial, PAHs constituted only a fraction of the chromatographic features resolved within solvent extracts of the material when analyzed by comprehensive high-resolution two-dimensional gas chromatography.^2^ No phthalates have been detected.^2^ However, several other tentatively identified compounds have included alkanes, alkenes, alkadienes,^2^ petroleum biomarkers,^2^ chemical additives (e.g., Irgafos 168, 1,3,5-tris(2,4-di-*t*-butylphenyl)phosphite;^2^ and Bumetrizole (Tinuvin-326), 2-(2-Hydroxy-3-*t*-butyl-5-methylphenyl)-5-chlorobenzotriazole^7^), their thermal breakdown products (e.g., 2,4-di-*t*-butylphenol),^2^ and metals (e.g., Ti, Zn, Mn, Co)^3,7,8^ as well as unknown compounds, demonstrating that the pyroplastic included a complex mixture of compounds, many with unknown bioactivity.

While the M/V *X-Press Pearl* disaster was a localized, acute release of pyroplastics, these forms of plastic have been documented on coastlines and in waterbodies globally (Figure S1). To date, the limited chemical analyses performed on beached pyroplastics unrelated to the M/V *X-Press Pearl* disaster have shown that these materials can be enriched in metals, PAHs, and phthalates.^9,10^ Pyroplastics are an emerging global contaminant thought to primarily enter the marine environment following fires at the wildland-urban interface and leaking of openly burned waste.^11−14^ Notably, open burning of plastic is now estimated to be the majority emission route for plastic waste to the environment.^15^

The toxicological concerns for burning plastic are not new, as emphasized by studies of the toxicity and chemistry of smoke and ash from residential and commercial fires,^16^ military burn pits,^17−19^ openly burned municipal waste,^13,20,21^ landfill fires,^22^ and fires at the wildland-urban interface^14,23^ as well as firewater runoff^24,25^ (the contaminated water produced during firefighting). However, these studies have not investigated the bioactivity of burnt plastic that remained after the fires were extinguished; their focus has largely been on aerosols and their impacts on air quality and human health. Similarly, despite their documented presence globally and unlike other plastic debris,^26^ the bioactivity of any pyroplastic is yet to be assessed. Not only is an assessment of potential toxicity necessary for making a hazardous waste determination of the spilled plastic,^5^ but there is also a need to measure its bioactivity owing to the friability of the pyroplastic, the elevated amounts of PAHs and other chemicals that can be associated with the pyroplastic, and the recognized “Trojan horse” effect for microplastic and nanoplastics to leach chemical pollutants to biota upon exposure (e.g., ingestion).^27−30^

Reporter bioassays have been a valuable method for determining the bioactivity of a chemical or complex mixture. Targeted bioassays have been used to screen extracts and leachates from consumer plastics,^31−34^ plastic photoproducts,^35^ weathered plastics,^26^ and combustion-derived particulate matter and ash,^23^ Measurements have primarily focused on the activation of the aryl hydrocarbon receptor (AhR), estrogen receptor (ER), androgen receptor (AR), pregnane X receptor (PXR), peroxisome proliferator-activated receptors (PPAR), and markers for oxidative stress (NRF2). Though targeted bioassays have been valuable, high-throughput, nontargeted screens of over 50 end points using the Attagene FACTORIAL platform can provide a more comprehensive assessment of bioactivity, capable of assigning chemicals and complex mixtures to specific modes of action.^36−43^ Additionally, as part of the United States Environmental Protection Agency (EPA) ToxCast program, the FACTORIAL platform has been used to evaluate more than 3000 chemicals, making it possible to compare bioactivities against an extensive database of diverse compounds.^39,44^ Moreover, variations of the platform (i.e., EcoTox and AquaTox) enable a harmonized cross-species assessment of endocrine and metabolic disruption upon chemical exposure for humans and wildlife (mouse, zebrafish, medaka, rainbow trout, chicken, frog, and turtle).^45,46^ Having such capabilities is valuable to addressing the potential ecotoxicity of pyroplastics. Recent work uncovering the acute toxicity of N-(1,3-dimethylbutyl)-*N*′-phenyl-p-phenylenediamine (6PPD) and its oxidized form (6PPD-quinone) to select salmonids and not others emphasizes the need to assay across phylogenetically separated species when assessing the potential ecotoxicity of plastic-associated chemicals.^47^ This is particularly needed for the M/V *X-Press Pearl* disaster as Sri Lankan fisheries rely on numerous, diverse fish species for sustenance.^48^

Herein, three FACTORIAL bioassays were used to assess the bioactivity of solvent extracts of white nurdles and pyroplastic collected within days of, and 242 days after, the M/V *X-Press Pearl* disaster. In total, the activities of 70 end points were measured, assessing the induction of human transcription factors and nuclear receptors related to biotransformation, lipid metabolism, the endocrine system, immunity, and cell stress, differentiation, and growth. Additionally, the AquaTox bioassay assessed 6 to 7 end points for endocrine and lipid metabolic function for each of three phylogenetically distinct fish species. Our findings provide needed information to guide long-term monitoring efforts, make hazard assessments of the spilled material, and direct further research on pyroplastics, an emerging global contaminant.

## Materials and Methods

### Sample Collection

Spilled plastics from the M/V *X-Press Pearl* disaster were collected from Pamunugama Beach, Sri Lanka, on May 25, 2021 (5 days after the fire began), and stray plastic related to the spill was collected from Sarakkuwa Beach, Sri Lanka, on January 17, 2022 (eight months after the spill). The two beaches are ∼2 km apart and were some of the closest shorelines to the ship. The recovered plastic was shipped to the Woods Hole Oceanographic Institution (Woods Hole, MA, USA) and stored at 4 °C as collected. All plastic was manipulated using solvent-rinsed stainless-steel tweezers. The material was visually sorted according to the categories operationally defined by de Vos et al.^2^ and James et al.,^6^ (i) white nurdles, (ii) burnt plastic, and (iii) excised pieces of combustion remnant (Figure 1). Samples from each category were previously analyzed for their PAH content.^5^ Orange and gray nurdles were not assayed because of limited sample quantity, and previous assessments demonstrated that their PAH composition reflected that of burnt plastic pieces.^5^ All plastic subsamples were visually consistent in form and color with that of previous reports for materials from the spill and cargo on board the ship (Table S1).

To provide a contrast to the complexity of the white nurdles and pyroplastics released during M/V *X-Press Pearl* spill, polyethylene nurdles from the M/V CMA CGA *Bianca* spill were also analyzed. These nurdles were released without exposure to additional chemicals from the ship or transformed by heat and combustion. Nurdles from the M/V CMA CGA *Bianca* pellet spill were graciously provided by Professor Mark Benfield (Louisiana State University). The nurdles were collected on August 13, 2020 (11 days after the spill) from the riverbank area of Chalmette Battlefield in New Orleans, Louisiana, USA.

### Solvent Extracts

Solvent extracts were prepared in triplicate by incubating ten nurdles or their equivalent mass of plastic in 5 mL (∼45 mg/mL) analytical grade dichloromethane (DCM) for 24 h at room temperature in combusted borosilicate glass vials with PTFE/F217 lined caps. DCM was used as a solvent because it would provide parity with previous chemical analyses conducted on the spilled plastic,^2,5^ it is commonly used to prepare extracts from combustion-derived plastics and materials for bioassays,^18,49−52^ and many classes of organic compounds, including hydrocarbons, are readily soluble in it. In terms of environmental relevance, the 24 h extraction by DCM likely constitutes a “worst-case” scenario in which nearly all hydrophobic contaminants are extracted. Additionally, they likely present some semblance to the loading that marine organisms may experience given the greater partitioning behavior of their stomach contents. For example, the hydrophobic oils within the stomach contents of marine birds can facilitate the substantial partitioning of hydrophobic contaminants from ingested plastic into the stomach contents within 24 h.^30^ After extraction, half of the DCM extract (2.5 mL) was blown to dryness under a gentle stream of nitrogen at room temperature and reconstituted in 100 μL of molecular biology grade dimethyl sulfoxide (DMSO). An extraction blank without plastic was also prepared. Specifics of each extract are provided in Table S1.

### TF-, NR-, and AquaTox-FACTORIAL Bioassays

DMSO-reconstituted DCM extracts were shipped to Attagene, Inc. (Morrisville, NC, USA) for testing by their TF-FACTORIAL (45 TF specific reporters), NR-FACTORIAL (24 human NRs) assays (previously named cis- and trans-FACTORIAL assays, respectively), and AquaTox-FACTORIAL (6 human NRs and 19 fish NRs).^38,39^ The assays use HepG2 cells to assess the activity of endogenous transcription factors (TF assay) or transfected hybrid proteins consisting of a yeast GAL4 DNA binding domain and ligand-binding domain of the human nuclear receptors (NR assay) or fish nuclear receptors (AquaTox assay). These multiplexed assays comprised 89 different measured end points related to cell stress, endocrine activity, growth and differentiation, immunity, and lipid, xenobiotic, and general metabolism.^43^ Extracts were tested at a maximum concentration of 3 μL DMSO extract/mL cell culture medium for 24 h. This concentration equates to the extractable content from ∼4 mg of spilled plastic (∼20% of the mass of a nurdle). Final DMSO concentrations were 0.3% (v/v). Five to six technical replicates of DMSO solvent controls matched to the DMSO concentration of the extracts were run with each sample set. Each extract was run as three technical replicates in Dulbecco’s Modified Eagle Medium (DMEM) containing 1% charcoal-stripped fetal bovine serum (FBS). The pyroplastics were evaluated by each FACTORIAL assay twice: the first at the maximum tested concentration for each of three extracts prepared from three independent sets of plastic, and the second as a 6-point serial dilution from the maximum tested concentration for a single representative extract. Each assay format was run once.

#### TF-FACTORIAL Assay

HepG2 cells were transfected with TF-FACTORIAL reporter library (46 TF-specific reporter plasmids and seven control reporters) using TransIT-LT1 transfection reagent according to the manufacturer’s protocol (Mirus). Transfected cells were plated into 12-well plates (3 × 10^5^/well), incubated for 24 h in their growth medium, washed, and treated with samples for 24 h in assay media (DMEM with 1% charcoal-stripped FBS). Cells were collected and processed.

#### NR-FACTORIAL Assay

HepG2 cells were transfected with NR-FACTORIAL library (25 GAL4-NR expression vector and corresponding reporter plasmid pairs) using TransIT-LT1 transfection reagent according to the manufacturer’s protocol (Mirus). Each pair of GAL4-NR/reporter was transfected separately to avoid cross-reactivity. Transfected cells were pooled together and plated into 12-well plates (3 × 10^5^/well), incubated for 24 h in growth media, washed and treated with tested samples for 24 h in assay medium (DMEM with 1% charcoal-stripped FBS). Cells were collected and processed.

#### AquaTox-FACTORIAL Assay

HepG2 cells were transfected with AquaTox-FACTORIAL library (25 GAL4-NR expression vector and corresponding reporter plasmid pairs) using TransIT-LT1 transfection reagent according to manufacturer’s protocol (Mirus). Each pair of GAL4-NR/reporter was transfected separately to avoid cross-reactivity. Transfected cells were pooled together and plated into 12-well plates (3 × 10^5^/well), incubated for 24 h in growth media, washed and treated with tested samples for 24 h in assay medium (DMEM with 1% charcoal-stripped FBS). Cells were collected and processed.

#### Sample Processing

Total RNA was isolated using PureLink Pro 96 total RNA Purification Kit (ThermoFisher). Reporter RNA was amplified by reverse-transcription polymerase chain reaction (RT-PCR) using a single pair of common primers. PCR fragments were labeled with fluorescent markers, and cut with *Hpa* I restriction enzyme, generating reporter-specific sizes of labeled DNA fragments that were quantitatively assayed by capillary electrophoresis using a Genetic Analyzer 3500xl. Bioassay responses were expressed as fold-induction relative to the DMSO control by dividing the treated cells’ average technical replicate expression by the average technical replicate expression of the appropriate DMSO control. Activation of an end point was operationally defined as requiring more than 1.5-fold induction across the two independently run assay formats and having a defined dose–response curve. All activities of an extraction blank were below the operationally defined induction cutoff (**Tables S2–S3**), and all positive control compounds activated receptors as expected (Table S4).

### Statistical Analysis

Statistical analyses were conducted using GraphPad Prism 10.2.3 (347). Data are presented as mean ± standard deviation (*n* = sample size). When appropriate, either parametric or nonparametric tests were used to compare groups. Groups were considered significantly different for a *p* value less than 0.05. EC_50_ concentrations and their asymmetrical 95% confidence intervals were calculated by fitting a three-parameter dose–response curve, . For the dose–response curves, the concentration was defined as the mass of extractable content per volume of cell culture medium used in the assay. Sample sizes and statistical tests are included in the text and figure captions where appropriate.

## Results

Chemicals associated with the spilled plastic may leach from the material over time once in the environment and following ingestion. To assess the amount of chemicals associated with the spilled plastic, the plastic was extracted with DCM. This slightly polar solvent readily dissolves petroleum-like hydrocarbons and other hydrophobic compounds typically associated with plastic found in the environment. DCM extractable contents for the white nurdles, burnt plastic, and combustion remnant pieces that first washed ashore on May 25, 2021, were 3 ± 4 mg/g plastic (*n* = 3), 24 ± 2 mg/g plastic (*n* = 3), and 88 ± 2 mg/g plastic (*n* = 3), respectively (Table S1). Comparatively, the DCM extractable content for the white nurdles and burnt plastic collected on January 17, 2022, 8 months after the spill, appeared relatively unchanged (unpaired *t* test with Welch’s correction; *p* value >0.05) with values of 5 ± 3 mg/g plastic (*n* = 3) and 19 ± 2 mg/g plastic (*n* = 3), respectively (Table S1).

To understand many of the biological pathways that could be affected by the complex mixture of plastic-associated chemicals, the solvent extracts from the spilled plastic were screened for their bioactivity using several FACTORIAL bioassays (TF, NR, and AquaTox). In total, across the three different bioassays, the activity of 70 human transcription factor response elements and nuclear receptors and 6 to 7 nuclear receptors for each of three phylogenetically distinct fish species were measured in response to the solvent extracts from white nurdles, burnt plastic, and combustion remnant pieces.

### Extracts of the Pyroplastic That First Washed Ashore on May 25, 2021, Activated Human Transcription Factors and Nuclear Receptors for Metabolic, Endocrine, and Cell Stress, Growth, and Differentiation Processes

**Figure 2:**
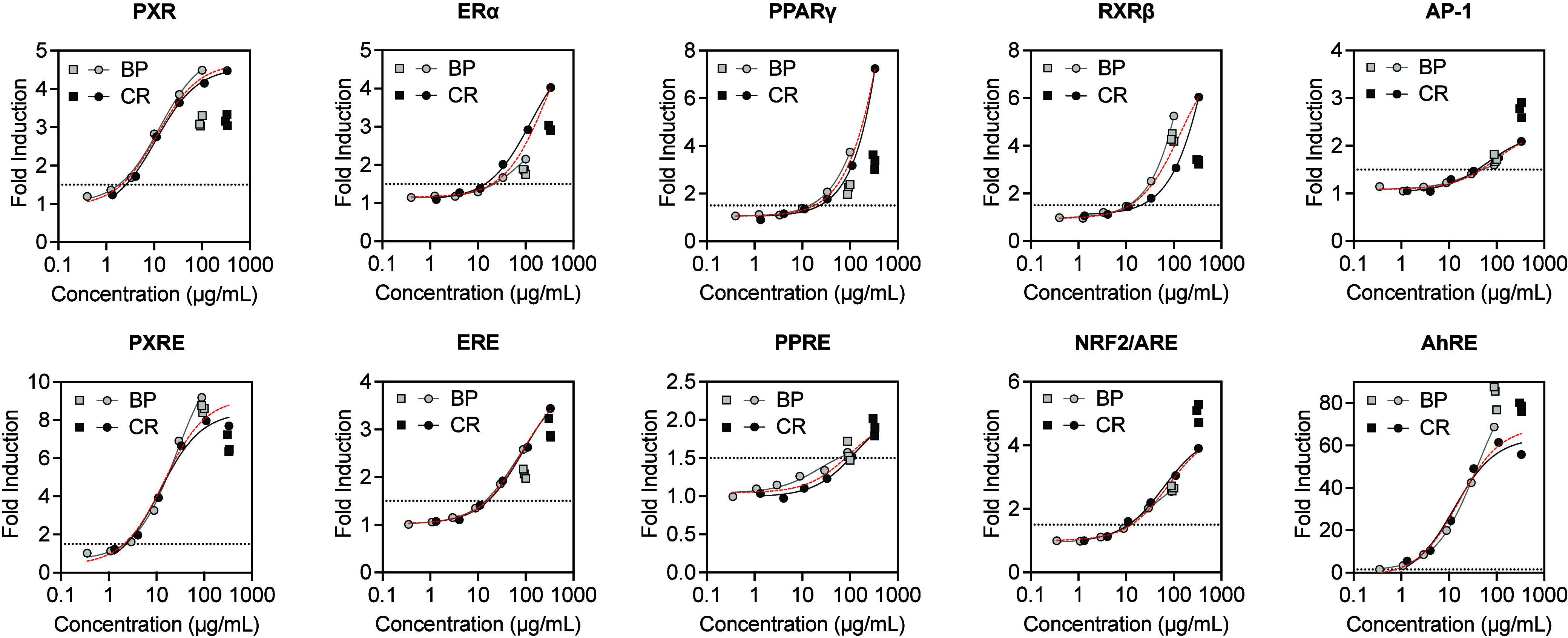
Human nuclear receptors and transcription factors activated by the pyroplastics. The dose–response activity of the NR- and TF- FACTORIAL end points for the solvent extracts of the burnt plastic (BP) and combustion remnant (CR) pieces collected on May 25, 2021. Data points are shown for the two FACTORIAL assay measurements: the first at the maximum tested concentration for each of three extracts prepared from three independent sets of plastic (squares), and the second as a serial dilution from the maximum tested concentration for a single representative extract from those previously evaluated (circles). Solid gray and black lines indicate the dose–response curves for the burnt plastic and combustion remnant, respectively. Dashed lines in red indicate the dose–response curve when values for the burnt plastic and combustion remnant were combined. Dotted lines indicate the operationally defined 1.5-fold induction criteria for activation. Concentration is presented as the mass of DCM extractable material per volume cell culture medium.

Bioactivity varied according to the type of spilled plastic. First, a single extract concentration was tested to semiquantitatively assess the variability in bioactivity within a sample type (e.g., white nurdle, burnt plastic, and combustion remnant). Subsequently, dose–response relationships were constructed for the bioactivity of the pyroplastics. Results were largely consistent across the three extracts prepared from three independent sets of plastic (Figure 2, Tables S5–S7). The coefficients of variation of the end points for the burnt plastic and combustion remnant ranged from 0.7 to 16.9% with a mean of 5.2% and 0.2% to 15.6% with a mean of 5.3%, respectively. As a result, one extract of each plastic type was used as a representative sample (Tables S6 and S7) for evaluating the dose–response activity of the pyroplastics. The variabilities for the activated end points between the two assay formats were within the reported biological variability of the assays.^38,40,53^

#### White Nurdles

Of the 45 human transcription factor response elements and 24 nuclear receptors tested for activity in the TF- and NR- FACTORIAL bioassays, the white nurdles activated only two end points above the operationally defined 1.5 fold-induction cutoff. At the concentration tested, only the aryl hydrocarbon receptor response element (AhRE) and the retinoid X receptor β (RXRβ) nuclear receptor exceeded the cutoff. The extracts induced average fold increases in activity of 1.76 ± 0.48 (*n* = 3) for AhRE and 1.84 ± 0.76 (*n* = 3) for RXRβ (Table S5). This amount of bioactivity was comparable (within the same order of magnitude) to that of polyethylene nurdles collected after the M/V CMA CGM *Bianca* containership plastic spill that happened along the banks of the Mississippi River in New Orleans, Louisiana, USA, in August 2020. Extracts of these nurdles induced average fold increases in activity of 3.79 ± 3.43 (*n* = 3) for AhRE; all other end points were below the operationally defined cutoff (Table S8). This spill was without fire, and so the source of the AhRE activity was attributed to hydrophobic organic contaminants from the Mississippi River that can associate with the nurdles.^54^ Additionally, assessment of the bioactivity of the white nurdles provided a pseudocontrol for evaluating the bioactivity of any chemical additives associated with the spilled plastic. The largely null results suggest the limited bioactivity of any chemical additives associated with the spilled plastics at the amounts present in the material and extractable by DCM. Thus, the bioactivity of the white nurdles appeared comparable to that of other polyethylene nurdles found in aquatic environments resulting from a containership spill. This finding also agreed with chemical analyses of their PAH content, which did not differ from that of other nurdles collected in the aquatic environment globally.^5^

#### Pyroplastics

The pyroplastics were more bioactive than the white nurdles, and activation trended with the amount of extractable material. The extracts from the burnt plastic and combustion remnant pieces activated several end points related to biotransformation, lipid, endocrine, and cell stress, growth, and differentiation processes (Figure 2, Tables S6, S7, S9, and S10). Specifically, the extracts activated the pregnane X receptor response element (PXRE) and its nuclear receptor (PXR), the estrogen receptor response element (ERE) and its receptor α (ERα), the peroxisome proliferator-activated receptor response element (PPRE) and its receptor γ (PPARγ), RXRβ, the nuclear erythroid-2 related factor 2-antioxidant response element (NRF2/ARE), the activator protein 1 (AP-1), and AhRE (Figure 2). The elevated activity of PXR, ERα, and PPARγ in the TF and NR assays for pyroplastic extracts suggested that active components of these extracts acted as direct ligands of PXR, ERα, and PPARγ. Activation of PXR was not surprising given its canonical role mediating the activity of many xenobiotic metabolizing enzymes.^55^ Direct ligand activation of ERα and PPARγ suggests compounds within the extracts may disrupt endocrine signaling and lipid metabolism.^56,57^ In fact, all three receptors have been implicated in the latter in response to obesogens.^57^

Several other end points demonstrated defined dose–response relationships that did not exceed the 1.5-fold induction cutoff operationally defined for activation or inconsistently exceeded the cutoff between the two independently run assay formats (Figure S2 and Tables S6, S7, S9, and S10). These end points included the liver X receptor α (LXRα), the constitutive androstane receptor (CAR), the peroxisome proliferator-activated receptor α (PPARα), the nuclear receptor related 1 (NURR1; also known as the nuclear receptor 4A2), the metal regulatory transcription factor 1 response element (MRE), the hypoxia-inducible factor-1α (HIF1α), the vitamin D receptor response element (VDRE), and the retinoic acid receptor-related orphan receptor response element (RORE). The activity of the liver X receptor family (direct repeat 4-binding proteins) response element (DR4/LXR) and the nuclear respiratory factor 1 (NRF1) activity were suppressed with increasing concentration of extractable material (Figure S2). The dose–response curves for AhRE, AP-1, CAR, ERE, ERα, HIF1α, LXRα, MRE, NRF2/ARE, NURR1, PPRE, PPARγ, PXRE, PXR, and VDRE showed tremendous concordance between the burnt plastic and combustion remnant with only minor deviations (i.e., the curves lined up on top of one another) (Figure 2 and Figure S2). The dose–response curves for DR4/LXR, PPARα, RORE, and RXRβ deviated substantially between the two plastic types (Figure 2 and Figure S2). The deviation was assessed qualitatively as the relative difference between the dose–response curves of the individual data sets and those of a dose–response curve for their combined data set.

### Bioactivity of Extracts from the Pyroplastic Collected Eight Months after the Spill Was Slightly Diminished

**Figure 3:**
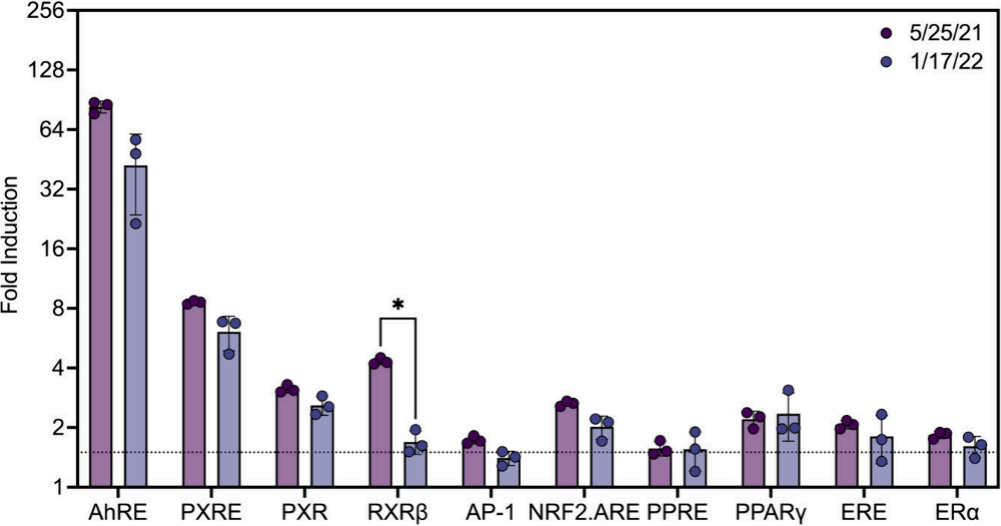
Activity of extracts from burnt plastic collected on May 25, 2021 (5/25/21) and January 17, 2022 (1/17/22). Statistical significance was evaluated by multiple unpaired Welch’s *t* tests corrected by the Holm-Šídák method for multiple comparisons. * denotes *p* value <0.05.

The bioactivity of white nurdles and burnt plastic collected eight months after the spill trended to lower values, and no additional end points were activated. On average, all end points in the TF- and NR- FACTORIAL bioassays were below the 1.5-fold induction criteria for extracts from white nurdles collected eight months after the spill on January 17, 2022 (Table S11). Additionally, this finding supports that the accumulation of any bioactive environmental contaminants on the spilled plastic was low compared to the amounts of compounds already present on the material when it first spilled. As for the burnt plastic, the induced average fold increase in activity trended lower; however, the activity of RXRβ was the only end point with a statistically significant reduction in activity (Figure 3, Table S12). Overall, the end points were more variable at the later time point, while the variability of the extractable mass was unchanged between time points. This difference suggested that while the amount of extractable material did not appear to change, there was a change in its composition during this period, which is supported by reported changes in the PAH content of the burnt plastic within this time frame.^5^

### Bioactivities of the Extracts from the Pyroplastic Were Species-Specific

**Figure 4:**
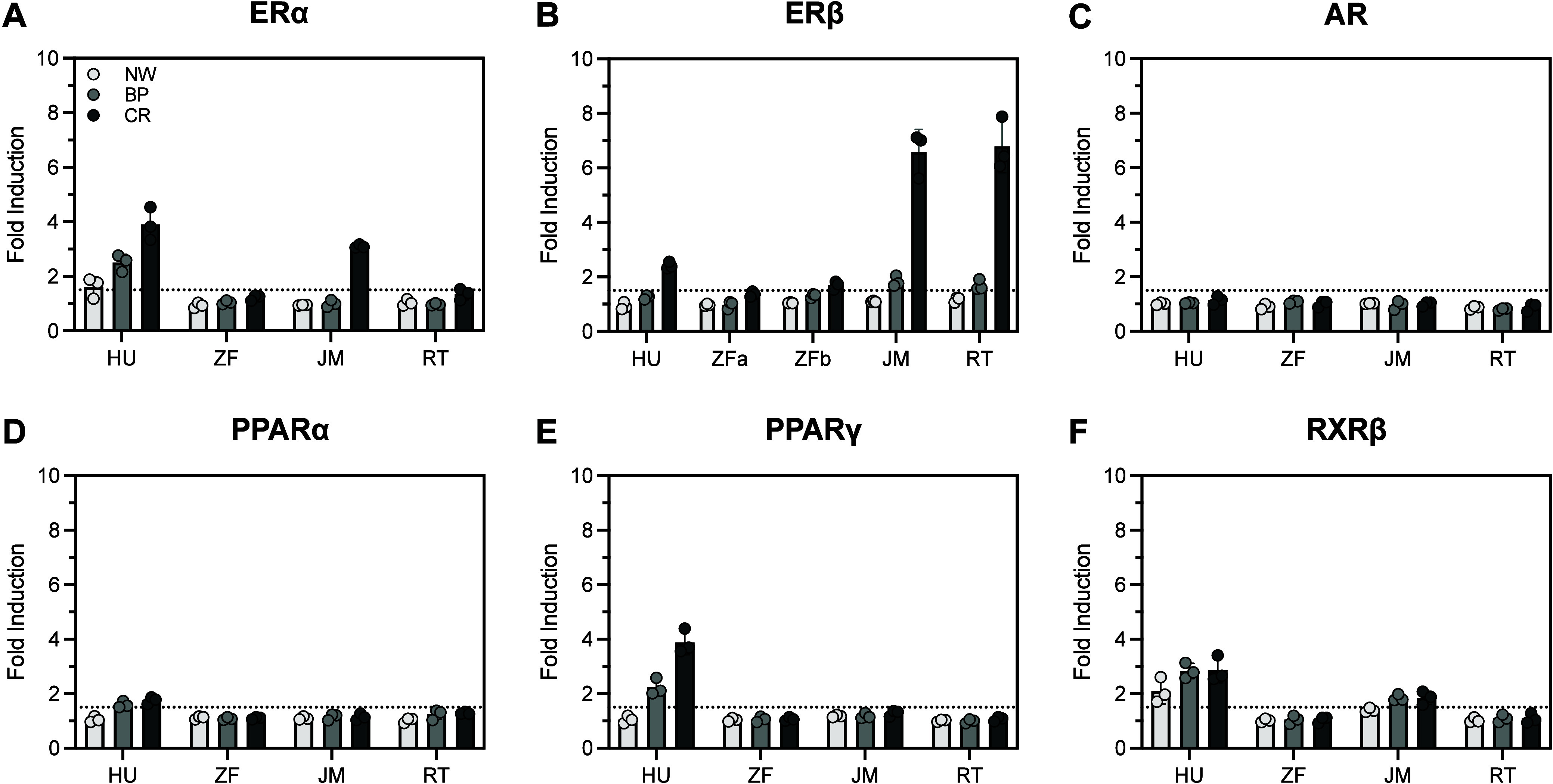
Bioactivity of AquaTox FACTORIAL end points for the solvent extracts of white nurdles (NW), burnt plastic (BP), and combustion remnant (CR) pieces collected on May 25, 2021. Dashed lines indicate the operationally defined 1.5-fold induction criteria for activation. Values for each extract are available in Tables S13–S15.

The AquaTox FACTORIAL bioassay revealed differences in nuclear receptor activation among fish species and between fish and human receptors. End points included the induction of species-specific estrogen receptors (ERα and ERβ), androgen receptors (AR), peroxisome proliferator-activated receptors (PPARα and PPARγ), and retinoid X receptors (RXRβ) for humans (HU), *Danio rerio* (zebrafish; ZF), *Oryzias latipes* (Japanese medaka; JM), and *Oncorhynchus mykiss* (rainbow trout; RT) that were expressed as GAL4-NR hybrid proteins in human HepG2 cells. Only end points for human receptors (ERα and RXRβ) were activated by extracts from the white nurdles. Fish ERα were largely unresponsive to the extracts from the spilled plastic (Figure 4A); only medaka ERα displayed activity above the 1.5-fold induction criteria in response to the combustion remnant extract (3.09 ± 0.06, n = 3). ERβ was the most sensitive to the pyroplastic extracts, and fish ERβ were more responsive to the pyroplastic extracts than human ERβ (Figure 4B). Medaka and rainbow trout ERβ were activated in response to the burnt plastic and combustion remnant extracts. Human ERβ and zebrafish ERβb expressed activity in response to the combustion remnant extract. None of the extracts elicited AR activity (Figure 4C). Fish PPARα and PPARγ were not activated by the plastic extracts, while the human PPARs were activated (Figure 4D-E). Human and medaka RXRβ showed activity in response to the pyroplastic extracts (Figure 4F). As with the TF- and NR- FACTORIAL bioassay results, the activity of the nurdles and pyroplastics collected eight months after the spill trended toward lower values (Figure S3, Tables S16 and S17). In conjunction with these semiquantitative results made at a single concentration, dose-activity measurements (Figure S4, Table S18) suggest the potential for the plastic-associated chemicals to disrupt fish estrogen signaling via different pathways depending on the fish species. In contrast, their ability to disrupt fish androgen and lipid metabolism via direct ligand-activated pathways is unlikely.

## Discussion

### Sources of Bioactivity

The FACTORIAL profiles suggest that the complex mixture of PAHs (and other compounds) within the pyroplastic extracts reflected that of a single PAH. Several PAHs–especially those that are possible or known human carcinogens–have been screened by the FACTORIAL bioassays as part of the ToxCast program and within Attagene’s FACTORIAL database. Profiles of the burnt plastic and combustion remnant were very similar to those of benzo[*b*]fluoranthene (BbF) and benzo[*k*]fluoranthene (BkF) (similarity score >0.96; 0.74 μmol/L; TF-FACTORIAL profile). Additionally, the TF-FACTORIAL profiles of the pyroplastic differed from those of other possible or known human carcinogenic PAHs, including benz[*a*]anthracene (BaA), chrysene (C0), benzo[*a*]pyrene (BaP), and indeno[1,2,3-*cd*]pyrene (IND). The AquaTox profiles also reflected this result, showing comparable similarity to BbF and not to BaP and BaA (Figures S5–S8) except for their ERβ activation, which was more akin to BaP and BaA than BbF. Notably, none of the single PAH compounds mentioned above activated PXRE/PXR and RXRβ and few activated PPRE/PPARγ while the pyroplastics robustly activated these end points (Figure 2, Figures S5–S8). These results suggest that other compounds in the complex chemical mixture were the cause of their activity.

From previous chemical analyses of the pyroplastics, the relative abundances of BaA, C0, BbF, BaP, and IND were comparable, and BkF was much less abundant than the others.^5^ Thus, the FACTORIAL profiles of the pyroplastics are unlikely to reflect the additive sum of the profiles for the individual compounds within the extracts. At first glance, this outcome aligns with evidence showing that complex mixtures of PAHs behave differently than single PAHs from a toxicological standpoint.^58,59^ Yet, in contradiction to this, the pyroplastic extracts primarily reflected profiles of a single PAH, but for a profile of a single PAH at an ∼10-fold greater concentration than is estimated to have been in the extract. Future work should investigate the FACTORIAL profiles of PAH mixtures to better interpret the contributions of each chemical component to the overall toxic potential of PAH complex mixtures that more represent real-world samples.

The findings reflect and expand on those made for other types of combustion-derived material (e.g., PAHs). Extracts of ash collected from forest fires at the wildland-urban interface were assessed for their AhR, ER, AR, interleukin-8, and cyclooxygenase-2 (COX-2) activity,^23^ which largely reflected the bioactivity of common combustion-derived chemicals of concern. There were some indications that other compounds contributed to the total bioactivity. However, their extent of activation was no more than that of the more common chemicals from an AhR activation standpoint. The FACTORIAL profiling of the pyroplastics for a much larger number of end points suggests a similar conclusion–the bioactivity reflected that of common combustion-derived chemicals of concern (i.e., PAHs). Nonetheless, while the FACTORIAL platform provides a valuable screen for bioactivity, an ensemble of measures (e.g., transcriptomics and other methods) is necessary to fully understand the potential modes of toxicity for a contaminant.

Moreover, the pyroplastics from the *X-Press Pearl* disaster were formed following the combustion of polyethylene. Other compounds of concern (e.g., dioxins, PFAS) can be formed during the combustion of halogenated and heteroatom-containing polymers.^13,20,21^ Thus, investigating pyroplastics from diverse plastics is necessary to more broadly confirm this similarity to other types of combustion-derived material.

### Bioactivity of the Uncollected Plastic

The findings suggest that nearly a year after the spill, the pyroplastics largely retained quantities and compositions of associated chemicals capable of eliciting bioactivity comparable to when the material first spilled. This outcome was not entirely unexpected given that polyethylene is used for the passive sampling of hydrophobic organic contaminants in the environment,^60^ i.e., partitioning between seawater and polyethylene skews toward greater amounts in polyethylene.^61^ With that in mind, the spilled plastic can accumulate and become enriched in additional contaminants from the environment.^62^ Continued monitoring of any uncollected plastic will be necessary to ascertain the extent to which its bioactivity profile and chemical complexity deviate from those when it first spilled over more extended periods in the environment.

### Potential Ecotoxicity of the Pyroplastics

Within the first few weeks of the M/V *X-Press Pearl* disaster, the spilled pyroplastics were expected to differentially impact wildlife because of their wide range of morphologies and physical properties.^2^ The AquaTox results expand upon this point. The data indicate that the potential toxicological harm from the plastic-associated chemicals will also be heterogeneous because of species-specific effects. In other words, fish species of comparable size (i.e., capable of ingesting similarly sized pyroplastics) can be expected to respond differently to the complex mixture of chemicals that leach from the material. This finding also likely translates to other taxonomic classes (e.g., birds). While, in hindsight, this conclusion may appear evident to those versed in comparative toxicology,^63,64^ during the environmental crisis of the spill, it was likely not at the forefront of concern. Instead, responders simply needed to know whether any chemicals associated with the plastic had toxicological potential. This point, however, is significant for monitoring programs and suggests the need to follow multiple phylogenetically distinct species within the same taxonomic class and use multiple end point measurements to best capture and assess potential harm.

### Adverse Outcome Pathways Related to the Activated End Points

Identifying pathway-based bioactivity in the samples can inform the potential hazards of exposure to chemicals associated with the pyroplastics. The adverse outcome pathway framework aims to connect *in vitro* pathway-based bioactivity (e.g., AhR activation) with organismal-level responses and adverse outcomes (e.g., cardiotoxicity).^65,66^ Adverse outcome pathways have been defined on AOP-Wiki^67^ for several of the activated end points, including PXR, AhR, ER, PPAR, and NRF2, while others (AP-1 and RXR) have yet to be established. The most developed adverse outcome pathways are for AhR and ER, whereby their activation has been connected to early mortality, several cancers, preeclampsia, cognitive decline, liver fibrosis and steatosis, and reproductive dysfunction. Activation of PPARα and PPARγ have adverse outcome pathways resulting in vascular disruption, obesity, liver steatosis, cancers, and reproductive dysfunction. The adverse outcome pathways associated with PXR and NRF2 are more nascent than the others; their activation includes liver steatosis and vascular disruption. Having identified potential upstream molecular initiating events with the FACTORIAL bioassays, future work should focus on hypothesis-driven, *in vivo* measures of tangential and downstream key events within these pathways to further guide risk assessment of pyroplastics.

## Conclusions

At the time of the spill, ∼1680 tons of plastic debris was released, of which a sizable portion was burned. By June 2021, ∼1610 tons of plastic, debris, and contaminated sand had been recovered and has since remained siloed in warehouses.^1^ Part of the prolonged containment of the waste has resulted from the uncertainty of its hazardousness and the methods for its appropriate disposal.^1^ From the FACTORIAL bioassays, it appears that the bioactivity of the chemicals associated with the pyroplastics largely reflects that of presumed and recognized carcinogenic PAHs, specifically BbF and BkF, despite being a complex mixture of thousands of compounds. This finding suggests that the material should be handled similarly to other combustion-derived residues (e.g., from biomass). Conversely, any chemicals associated with the white nurdles appear to pose a comparatively marginal threat, eliciting relatively minimal bioactivity at their expected concentrations. Nonetheless, the bioavailability of the associated chemicals, which controls their effective dosage, remains to be determined. For the stray pyroplastic still in the environment, continued monitoring is necessary. Pieces of pyroplastic collected nearly a year after the spill largely retained quantities and compositions of associated chemicals capable of eliciting bioactivity comparable to when they first spilled. As the material fragments into smaller pieces, other organisms will become susceptible to it, and other modes of toxicological action will likely arise (e.g., submicron-sized plastic particles loaded with relatively high levels of contaminants).^68,69^ With the detection of pyroplastics across much of the globe and recently in fish,^70^ further understanding of their toxicity is needed.

## Data Availability

All data is included in the manuscript and Supporting Information. Subsamples of the spilled plastic are available upon request.

## Supporting Information Available

Sightings of pyroplastics globally, dose–response curves of additional TF- and NR-FACTORIAL end points, AquaTox-FACTORIAL end points for burnt plastic pieces collected on May 25, 2021 and January 17, 2022, dose–response activity of the AquaTox-FACTORIAL end points for the pyroplastics, AquaTox-FACTORIAL dose–response profiles for ERα of BaA, BaP, BbF, AquaTox-FACTORIAL dose–response profiles for ERβ of BaA, BaP, BbF, AquaTox-FACTORIAL dose–response profiles for PPARγ of BaA, BaP, BbF, AquaTox-FACTORIAL dose–response profiles for RXRβ of BaA, BaP, BbF, and additional references (PDF)
Subsample details, TF-FACTORIAL and NR-FACTORIAL data for DCM blank, AquaTox-FACTORIAL data for DCM blank, AquaTox FACTORIAL controls, TF-FACTORIAL and NR-FACTORIAL data for NW 5/25/21, TF-FACTORIAL and NR-FACTORIAL data for BP 5/25/21, TF-FACTORIAL and NR-FACTORIAL data for CR 5/25/21, TF-FACTORIAL and NR-FACTORIAL data for other nurdle spill, TF-FACTORIAL and NR-FACTORIAL EC_50_ for BP 5/25/21, TF-FACTORIAL and NR-FACTORIAL EC_50_ for CR 5/25/21, TF-FACTORIAL and NR-FACTORIAL data for NW 1/17/22, TF-FACTORIAL and NR-FACTORIAL data for BP 1/17/22, AquaTox-FACTORIAL data for NW 5/25/21, AquaTox-FACTORIAL data for BP 5/25/21, AquaTox-FACTORIAL data for CR 5/25/21, AquaTox-FACTORIAL data for NW 1/17/22, AquaTox-FACTORIAL data for BP 1/17/22, and AquaTOX-FACTORIAL EC_50_ for CR 5/25/21 (XLSX)
