# Supplementary material for: Burnt Plastic (Pyroplastic) from the M/V X-Press Pearl Ship Fire and Plastic Spill Contain Compounds That Activate Endocrine and Metabolism-Related Human and Fish Transcription Factors
Source: Environ Health (Wash). 2024 Oct 30;3(1):91–101. doi: 10.1021/envhealth.4c00172 (PMC11744394; doi:10.1021/envhealth.4c00172)
Supplement: Supplementary file 1 — eh4c00172_si_001.pdf [file eh4c00172_si_001.pdf]

Supporting Information

Burnt plastic (pyroplastic) from the M/V *X-Press Pearl* ship fire and plastic spill contain compounds that activate endocrine and metabolism-related human and fish transcription factors

Bryan D. James[1,2,3]*, Alexander V. Medvedev[4], Lyubov A. Medvedeva[4], Elena Martsen[4], Kristen L. Gorman[4], Benjamin Lin[4], Sergei S. Makarov[4], Lihini I. Aluwihare[5], Asha de Vos[6,7], Christopher M. Reddy[1], Mark E. Hahn[2]

[1]Department of Marine Chemistry and Geochemistry, Woods Hole Oceanographic Institution, Woods Hole, Massachusetts, 02543, United States

[2]Department of Biology, Woods Hole Oceanographic Institution, Woods Hole, Massachusetts, 02543, United States

[3]Department of Chemical Engineering, Northeastern University, Boston, Massachusetts, 02115, United States

[4]Attagene, Research Triangle Park, Morrisville, North Carolina, 27709, United States

[5]Scripps Institution of Oceanography, University of California San Diego, La Jolla, California, 92093, United States

[6]Oceanswell, Colombo, 00500, Sri Lanka

[7]The Oceans Institute, University of Western Australia, Perth, 6009, Australia

*Corresponding author

Bryan D. James, Email: b.james@northeastern.edu

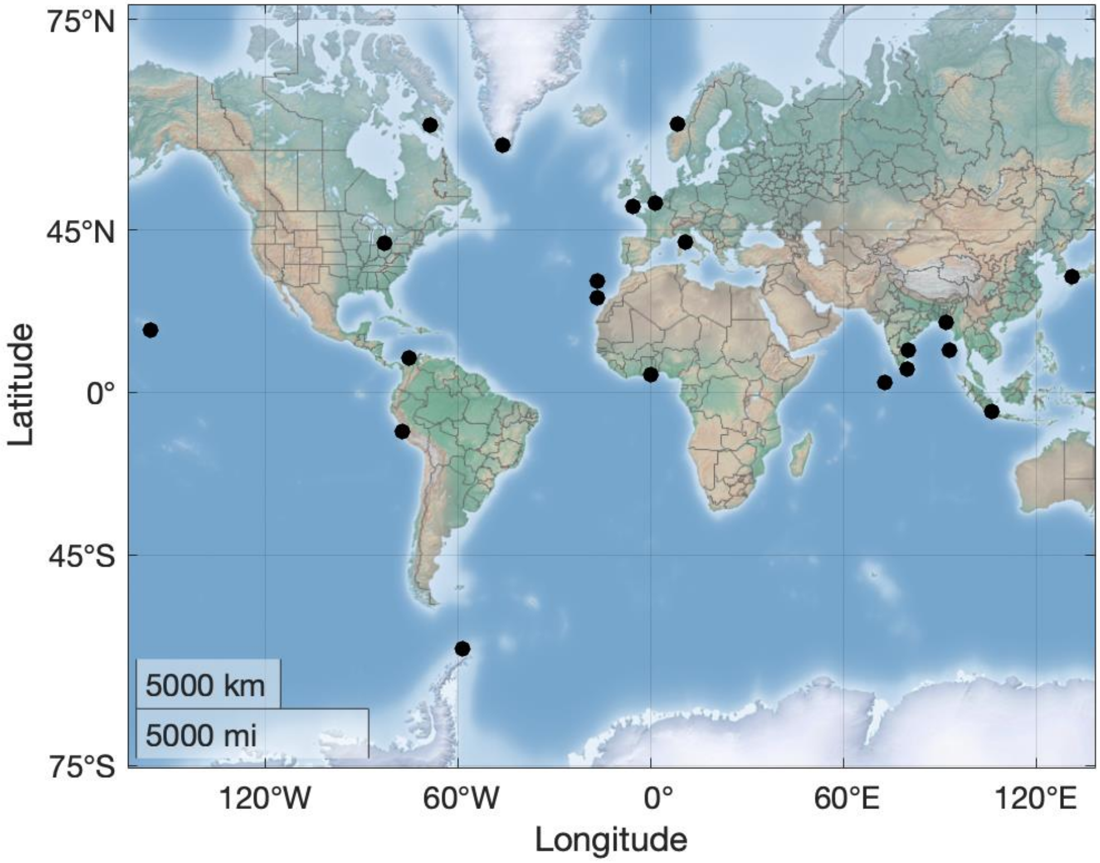

Figure S1. Reports of pyroplastic globally. Pyroplastics have been found on coastlines and in waterbodies in Africa,[1,2] Antarctica,[3] Asia,[4–10] Europe,[11–15] North America,[11,16,17] and South America.[18,19]

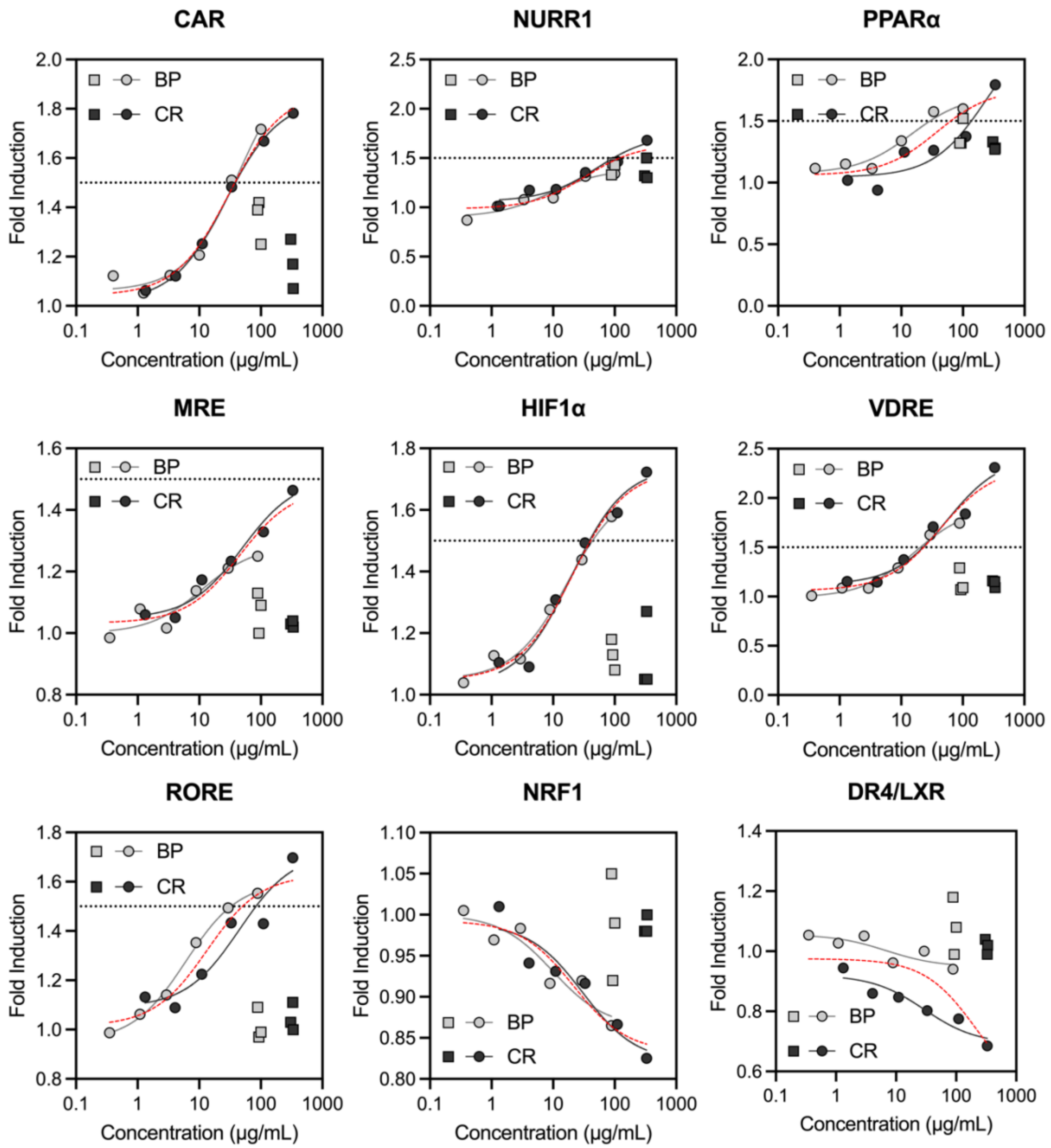

Figure S2. Human nuclear receptors and transcription factors with potential for activation by the pyroplastics. Dose-response activity of the NR- and TF- FACTORIAL end points for the solvent extracts of the burnt plastic (BP) and combustion remnant (CR) pieces collected on May 25, 2021. Data points are shown for the two FACTORIAL assay measurements: the first at the maximum tested concentration for each of three extracts prepared from three independent sets of plastic (squares), and the second as a serial dilution from the maximum tested concentration for a single representative extract from those previously evaluated (circles). Solid grey and black lines indicate the dose-response curves for the burnt plastic and combustion remnant, respectively. Dashed lines in red indicate the dose-response curve when values for the burnt plastic and combustion remnant were combined. Dotted lines indicate the operationally-defined 1.5-fold induction criteria for activation. Concentration is presented as the mass of DCM extractable material per volume cell culture medium.

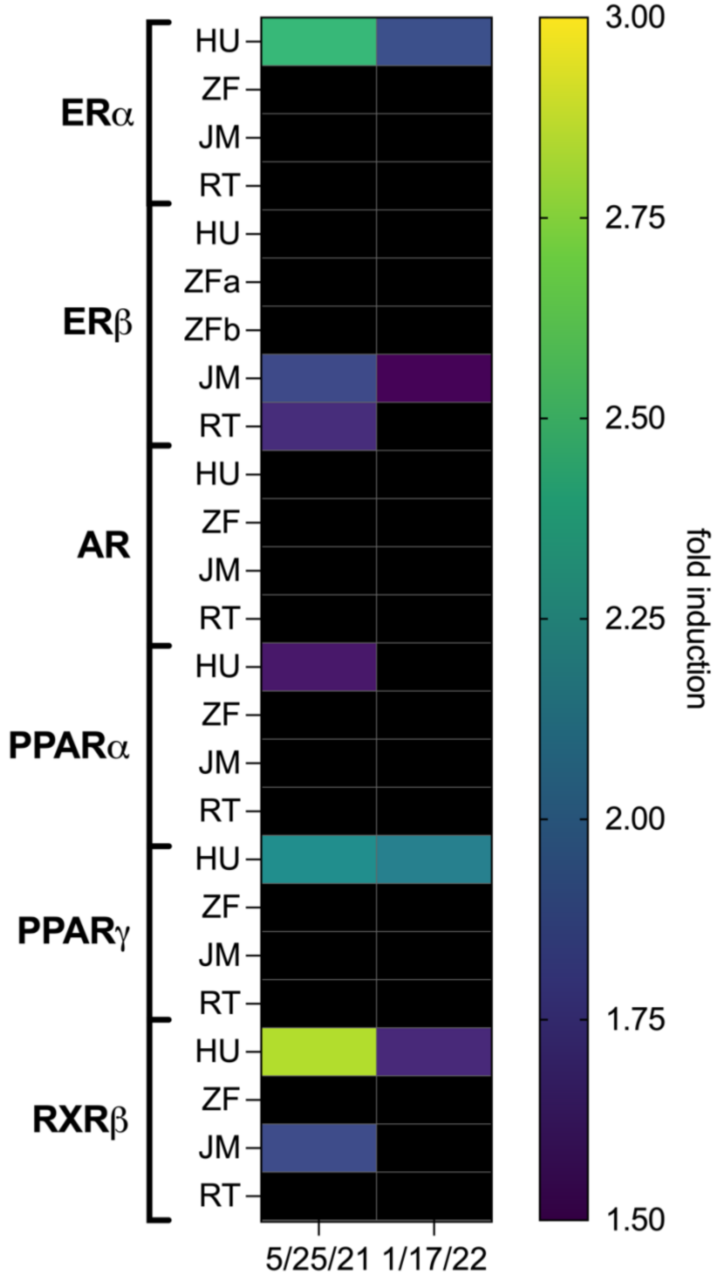

Figure S3. Activity of the AquaTox FACTORIAL end points for the extracts from the burnt plastic collected on May 25, 2021 (5/25/21) and January 17, 2022 (1/17/22). End points included the induction of species-specific estrogen receptors (ERα and ERβ), androgen receptors (AR), peroxisome proliferator-activated receptors (PPARα and PPARγ), and retinoid X receptors (RXRβ) for humans (HU), *Danio rerio* (zebrafish; ZF), *Oryzias latipes* (Japanese medaka; JM), and *Oncorhynchus mykiss* (rainbow trout; RT). Values for each extract are available in **Tables S14 and S17**. Cells in black have average values below the operationally defined 1.5-fold cut-off.

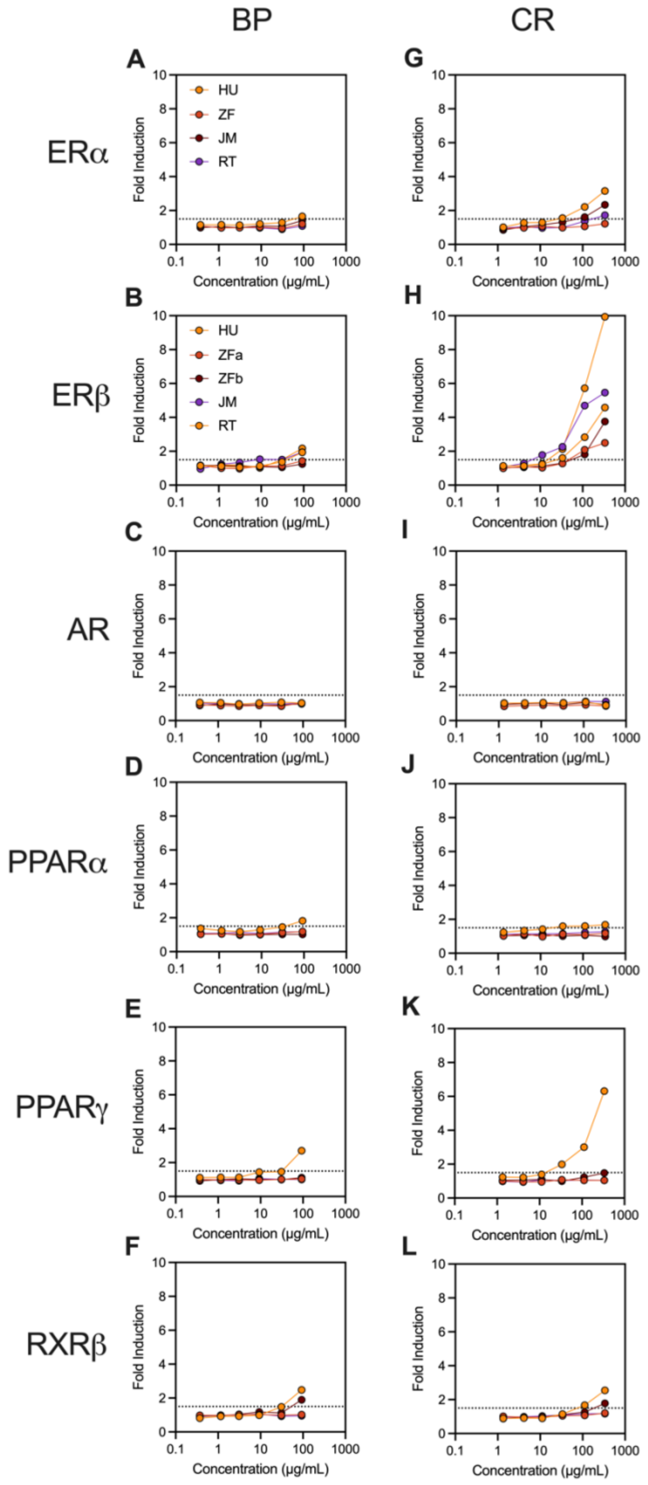

Figure S4. Dose-response activity of the AquaTox FACTORIAL end points for the extracts of the burnt plastic (A-F) and combustion remnant (G-L) pieces collected on May 25, 2021. Dashed lines indicate the operationally-defined 1.5-fold induction criteria for activation. Given the consistency across extracts, extracts BP1 and CR2 were assayed as representative of the sample sets. Concentration is presented as mass of DCM extractable content per volume cell culture medium.

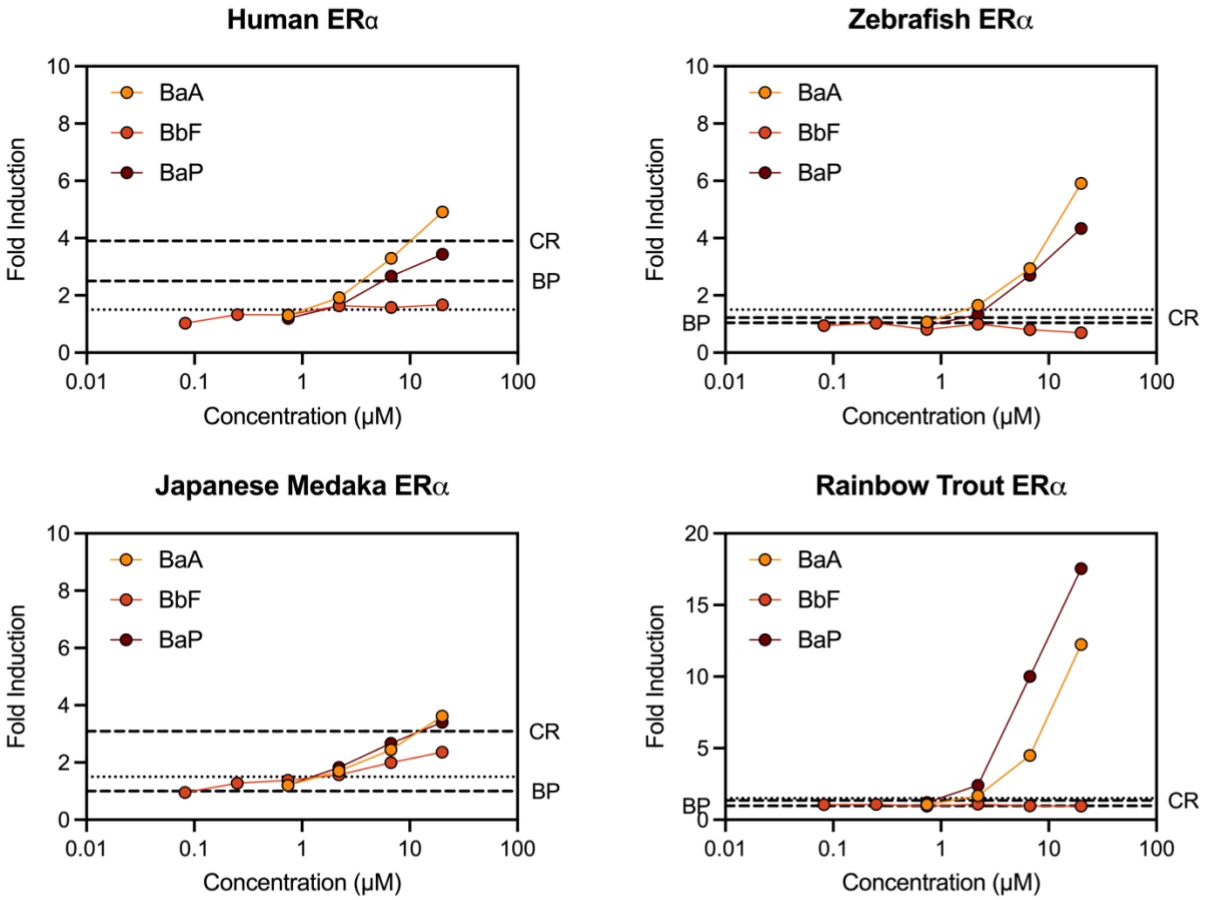

Figure S5. Comparison of AquaTox FACTORIAL dose-response profiles for ERα of BaA, BaP, BbF. Dotted lines indicate the operationally-defined 1.5 induction cut-off. Dashed lines indicate the average fold induction of the burnt plastic (BP) and combustion remnant (CR) tested at a concentration of 3 μL extract/mL cell culture medium.

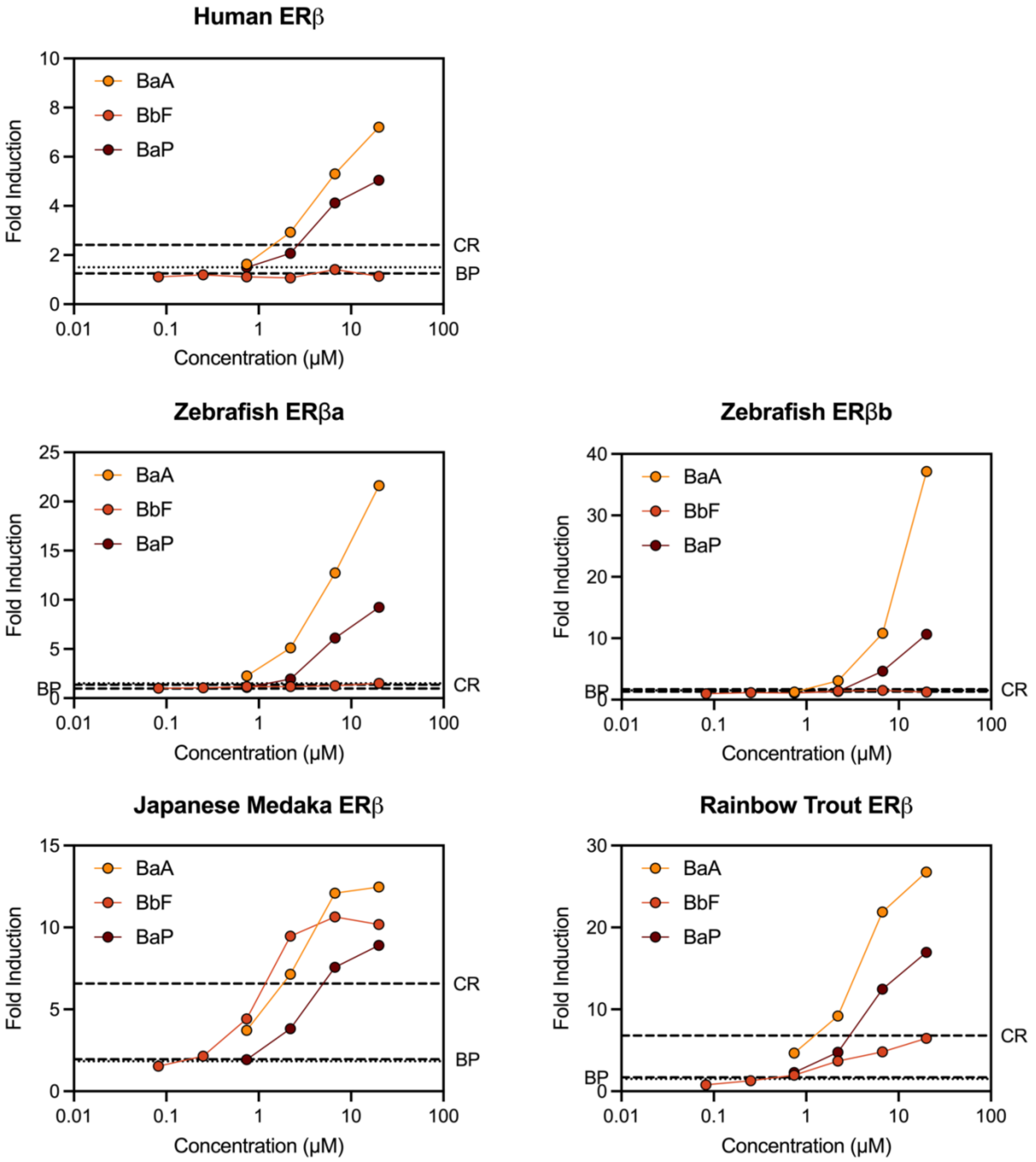

Figure S6. Comparison of AquaTox FACTORIAL dose-response profiles for ERβ of BaA, BaP, BbF. Dotted lines indicate the operationally-defined 1.5 induction cut-off. Dashed lines indicate the average fold induction of the burnt plastic (BP) and combustion remnant (CR) tested at a concentration of 3 μL extract/mL cell culture medium.

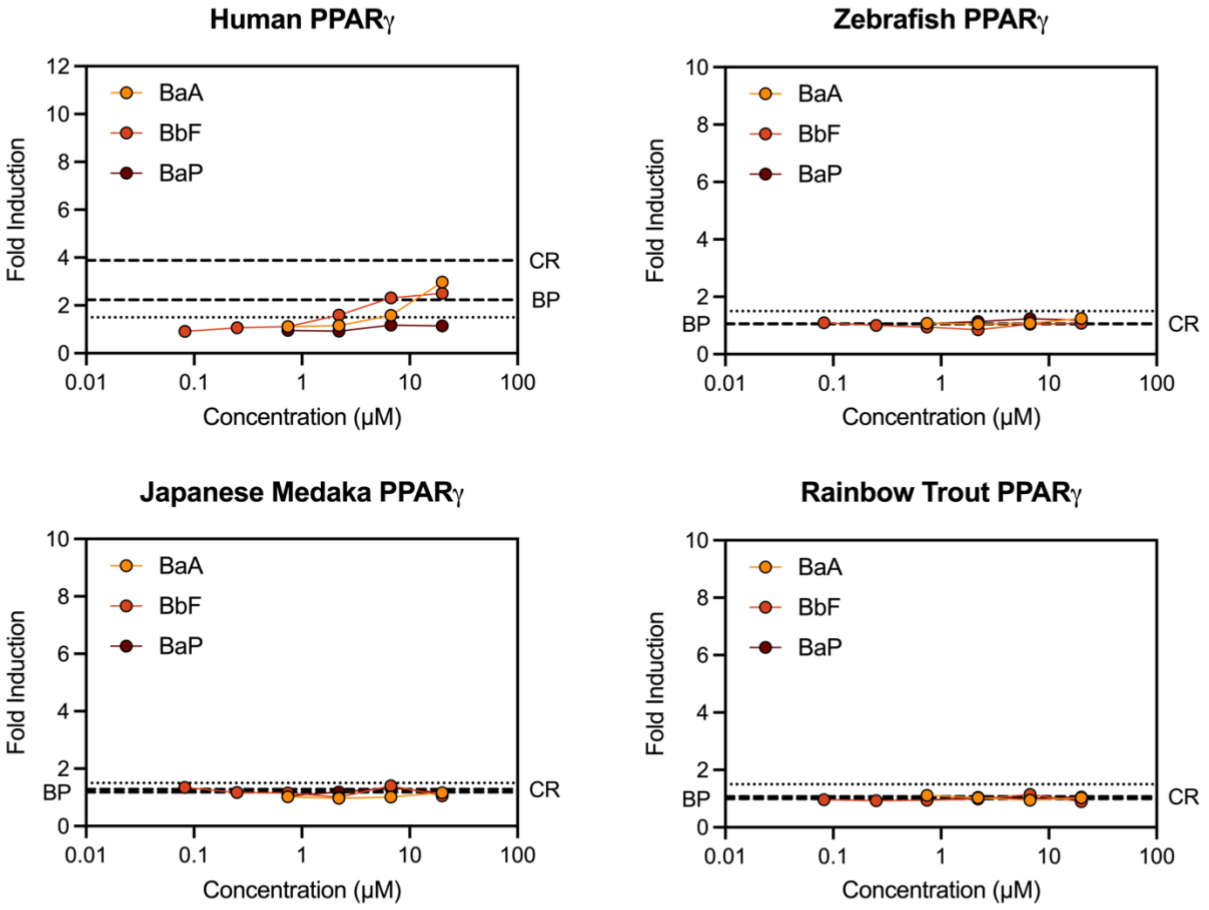

Figure S7. Comparison of AquaTox FACTORIAL dose-response profiles for PPARγ of BaA, BaP, BbF. Dotted lines indicate the operationally-defined 1.5 induction cut-off. Dashed lines indicate the average fold induction of the burnt plastic (BP) and combustion remnant (CR) tested at a concentration of 3 μL extract/mL cell culture medium.

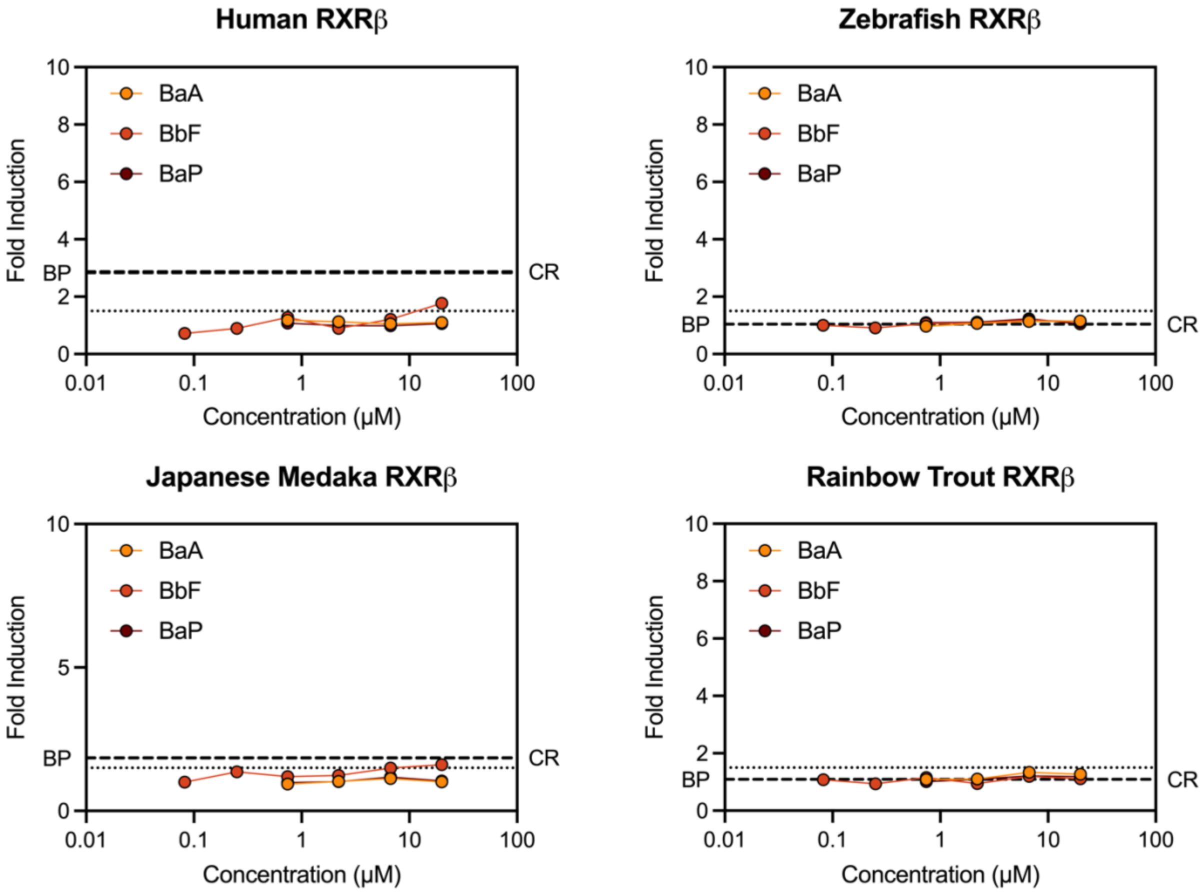

Figure S8. Comparison of AquaTox FACTORIAL dose-response profiles for RXRβ of BaA, BaP, BbF. Dotted lines indicate the operationally-defined 1.5 induction cut-off. Dashed lines indicate the average fold induction of the burnt plastic (BP) and combustion remnant (CR) tested at a concentration of 3 µL extract/mL cell culture medium.

References

(1) Adika, S. A.; Mahu, E.; Crane, R.; Marchant, R.; Montford, J.; Folorunsho, R.; Gordon, C. Microplastic Ingestion by Pelagic and Demersal Fish Species from the Eastern Central Atlantic Ocean, off the Coast of Ghana. *Marine Pollution Bulletin* **2020**, *153*, 110998. https://doi.org/10.1016/j.marpolbul.2020.110998.

(2) Domínguez-Hernández, C.; Vega-Moreno, D.; Villanova-Solano, C.; Hernández-Sánchez, C.; Lambre, M. E.; Hernández-Borges, J. Characterization of Pyroplastics from the North Atlantic. *Marine Pollution Bulletin* **2024**, *208*, 116960. https://doi.org/10.1016/j.marpolbul.2024.116960.

(3) Lozoya, J. P.; Rodríguez, M.; Azcune, G.; Lacerot, G.; Pérez-Parada, A.; Lenzi, J.; Rossi, F.; de Mello, F. T. Stranded Pellets in Fildes Peninsula (King George Island, Antarctica): New Evidence of Southern Ocean Connectivity. *Science of The Total Environment* **2022**, *838*, 155830. https://doi.org/10.1016/j.scitotenv.2022.155830.

(4) Furukuma, S. A Study of 'New Plastic Formations' Found in the Seto Inland Sea, Japan. *International Journal of Scientific and Research Publications (IJSRP)* **2021**, *11* (6), 185–188. https://doi.org/10.29322/IJSRP.11.06.2021.p11427.

(5) Furukuma, S.; Ellrich, J. A.; Ehlers, S. M. Frequent Observations of Novel Plastic Forms in the Ariho River Estuary, Honshu, Japan. *Science of The Total Environment* **2022**, *848*, 157638. https://doi.org/10.1016/j.scitotenv.2022.157638.

(6) Saliu, F.; Montano, S.; Garavaglia, M. G.; Lasagni, M.; Seveso, D.; Galli, P. Microplastic and Charred Microplastic in the Faafu Atoll, Maldives. *Marine Pollution Bulletin* **2018**, *136*, 464–471. https://doi.org/10.1016/j.marpolbul.2018.09.023.

(7) Goswami, P.; Bhadury, P. First Record of an Anthropocene Marker Plastiglomerate in Andaman Island, India. *Marine Pollution Bulletin* **2023**, *190*, 114802. https://doi.org/10.1016/j.marpolbul.2023.114802.

(8) Utami, D. A.; Reuning, L.; Schwark, L.; Friedrichs, G.; Dittmer, L.; Nurhidayati, A. U.; Al Fauzan, A.; Cahyarini, S. Y. Plastiglomerates from Uncontrolled Burning of Plastic Waste on Indonesian Beaches Contain High Contents of Organic Pollutants. *Scientific Reports* **2023**, *13* (1), 10383. https://doi.org/10.1038/s41598-023-37594-z.

(9) Gunasekaran, K.; Mghili, B.; De-la-Torre, G. E.; Sompongchaiyakul, P.; Rangel-Buitrago, N.; Wang, X.; Charoenpong, C. First Record of Plastiglomerates, Pyroplastics and Plasticrusts along the Beaches of Tamilnadu, Southeast Coast of India. *Marine Pollution Bulletin* **2024**, *205*, 116594. https://doi.org/10.1016/j.marpolbul.2024.116594.

(10) Rakib, Md. R. J.; De-la-Torre, G. E.; Jolly, Y. N.; Al Nahian, S.; Khan, N. I.; Idris, A. M. First Record of Plastiglomerate and Pyroplastic Pollution in the World's Longest Natural Beach. *Science of The Total Environment* **2023**, *891*, 164369. https://doi.org/10.1016/j.scitotenv.2023.164369.

(11) Turner, A.; Wallerstein, C.; Arnold, R.; Webb, D. Marine Pollution from Pyroplastics. *Science of The Total Environment* **2019**, *694*, 133610. https://doi.org/10.1016/j.scitotenv.2019.133610.

(12) Ellrich, J. A.; Ehlers, S. M. Field Observations in Pebble Beach Habitats Link Plastiglomerate to Pyroplastic via Pebble Clasts. *Marine Pollution Bulletin* **2022**, *174*, 113187. https://doi.org/10.1016/j.marpolbul.2021.113187.

(13) Ehlers, S. M.; Ellrich, J. A. First Record of 'Plasticrusts' and 'Pyroplastic' from the Mediterranean Sea. *Marine Pollution Bulletin* **2020**, *151*, 110845. https://doi.org/10.1016/j.marpolbul.2019.110845.

(14) Zardi, G. I.; Seuront, L.; Spilmont, N.; Froneman, P. W.; Nicastro, K. R. Leachates from Pyroplastics Alter the Behaviour of a Key Ecosystem Engineer. *Estuarine, Coastal and Shelf Science* **2024**, *301*, 108740. https://doi.org/10.1016/j.ecss.2024.108740.
(15) Cyvin, J. B.; Ervik, H.; Kveberg, A. A.; Hellevik, C. Macroplastic in Soil and Peat. A Case Study from the Remote Islands of Mausund and Froan Landscape Conservation Area, Norway; Implications for Coastal Cleanups and Biodiversity. *Science of The Total Environment* **2021**, *787*, 147547. https://doi.org/10.1016/j.scitotenv.2021.147547.
(16) Arturo, I. A.; Corcoran, P. L. Categorization of Plastic Debris on Sixty-Six Beaches of the Laurentian Great Lakes, North America. *Environmental Research Letters* **2022**, *17* (4), 045008. https://doi.org/10.1088/1748-9326/ac5714.
(17) Liboiron, M.; Zahara, A.; Hawkins, K.; Crespo, C.; de Moura Neves, B.; Wareham-Hayes, V.; Edinger, E.; Muise, C.; Walzak, M. J.; Sarazen, R.; Chidley, J.; Mills, C.; Watwood, L.; Arif, H.; Earles, E.; Pijogge, L.; Shirley, J.; Jacobs, J.; McCarney, P.; Charron, L. Abundance and Types of Plastic Pollution in Surface Waters in the Eastern Arctic (Inuit Nunangat) and the Case for Reconciliation Science. *Science of The Total Environment* **2021**, *782*, 146809. https://doi.org/10.1016/j.scitotenv.2021.146809.
(18) De-la-Torre, G. E.; Pizarro-Ortega, C. I.; Dioses-Salinas, D. C.; Rakib, Md. R. J.; Ramos, W.; Pretell, V.; Ribeiro, V. V.; Castro, Í. B.; Dobaradaran, S. First Record of Plastiglomerates, Pyroplastics, and Plasticrusts in South America. *Science of The Total Environment* **2022**, *833*, 155179. https://doi.org/10.1016/j.scitotenv.2022.155179.
(19) Rangel-Buitrago, N.; Ochoa, F. L.; Rodríguez, R. D. B.; Moreno, J. B.; Trilleras, J.; Arana, V. A.; Neal, W. J. Decoding Plastic Pollution in the Geological Record: A Baseline Study on the Caribbean Coast of Colombia, North South America. *Marine Pollution Bulletin* **2023**, *192*, 114993. https://doi.org/10.1016/j.marpolbul.2023.114993.
